# Selection within working memory impairs perceptual detection

**DOI:** 10.3758/s13423-022-02238-2

**Published:** 2023-01-03

**Authors:** Joaquín Macedo-Pascual, Almudena Capilla, Pablo Campo, José Antonio Hinojosa, Claudia Poch

**Affiliations:** 1 Departamento de Psicología Experimental, Procesos Cognitivos y Logopedia, Universidad Complutense de Madrid, Madrid, Spain; 2 Departamento de Psicología Biológica y de la Salud, Universidad Autónoma de Madrid, Madrid, Spain; 3 Departamento de Psicología Básica, Universidad Autónoma de Madrid, Madrid, Spain; 4 Instituto Pluridisciplinar, Universidad Complutense de Madrid, Madrid, Spain; 5 Centro de Investigación Nebrija en Cognición (CINC), Universidad Nebrija, Madrid, Spain; 6 Departamento de Educación, Universidad Nebrija, C. de Sta. Cruz de Marcenado, 27, 28015 Madrid, Spain

**Keywords:** Visual WM, Attention, Retro-cue, Alpha oscillations

## Abstract

There is broad consensus supporting the reciprocal influence of working memory (WM) and attention. Top-down mechanisms operate to cope with either environmental or internal demands. In that sense, it is possible to select an item within the contents of WM to endow it with prioritized access. Although evidence supports that maintaining an item in this privileged state does not rely on sustained visual attention, it is unknown whether selection within WM depends on perceptual attention. To answer this question, we recorded electrophysiological neural activity while participants performed a retro-cue task in which we inserted a detection task in the delay period after retro-cue presentation. Critically, the onset of to-be-detected near threshold stimuli was unpredictable, and thus, sustained perceptual spatial attention was needed to accomplish the detection task from the offset of the retro-cue. At a behavioral level, we found decreased visual detection when a WM representation was retro-cued. At a neural level, alpha oscillatory activity confirmed a spatial shift of attention to the retro-cued representation. We interpret the convergence of neural oscillations and behavioral data to point towards the theory that selection within WM could be accomplished through a perceptual attentional mechanism.

## Introduction

Working memory (WM) has been metaphorically defined as the sketchpad of conscious thought (Miller et al., 2018) that allows us to temporally hold and manipulate mental representations in order to accomplish an ongoing task (Cowan, 2022; Gazzaley & Nobre, 2012; Oberauer, 2019). Although there is enough consensus about a close link between attention and WM, the nature of this relation is not well established, partially due to the multidimensional concept of attention (Oberauer, 2019). The models of WM that emphasize the selective dimension of attention conceptualize WM as a top-down process that maintains mental representations in an active state in an analogous way to how attention operates on perceptual stimuli (Chun, 2011; Chun et al., 2011; D’Esposito & Postle, 2015; Kiyonaga & Egner, 2013). With this view, a key question in order to unravel the nature of the connection between WM and attention is to what extent the attentional mechanisms that determine what is encoded and maintained in WM are superimposed on those employed towards external stimuli. Answers to this question come from neuroimaging studies and behavioral data. Neuroimaging experiments comparing the neural substrates involved in spatial attention and mnemonic maintenance have found overlapping networks (Awh & Jonides, 2001; Ikkai & Curtis, 2011; Nee & Jonides, 2008; Nobre et al., 2004; Panichello & Buschman, 2021), proposing that the maintenance of WM representations is based on an attention-based rehearsal mechanism. In an analogous way, the oculomotor system seems to be implicated in the maintenance of WM representations (Pearson & Sahraie, 2003; Williams et al., 2013). In the same vein, behavioral data shows that spatial WM declines when a secondary task demands shifts of spatial attention to external locations (Awh et al., 1998; Van Der Stigchel et al., 2007). Similar to cited studies that find that diverting spatial attention from memory representations impairs spatial WM (Awh et al., 1998), other studies find an inverse trade-off between memory and perception, finding impaired perception of low-contrast stimuli when WM load is increased (Balestrieri et al., 2021; Konstantinou et al., 2014).

In apparent incongruency with the presented evidence supporting that WM maintenance is accomplished through perceptual attention, some studies have suggested that not all WM representations are maintained through perceptual attention (Gao et al., 2022; Hedge et al., 2015; Hollingworth & Maxcey-Richard, 2013; Rerko et al., 2014). In this sense, the representation in the focus of attention (FoA), defined as the item that through an attentional selective mechanism is conferred with a privileged state of accessibility (Larocque et al., 2014), would not be affected by a concurrent visual attentional task. The study of the interaction between visual attention and the internal FoA has been approached through two different types of paradigms. Some studies have used the object switch paradigm to study the impact of diverting perceptual spatial attention away from the to be updated representation, finding that the object switching cost remains invariable (Hedge et al., 2015). Therefore, these authors conclude that perceptual attention is not responsible for the advantage of updating the same memory item, and rather conceptualize the link between attention in the mnemonic and perceptual domains as a shared spatial priority map (Hedge et al., 2015; Van Der Stigchel et al., 2007). Studies using the retro-cue paradigm have found that inserting a perceptually demanding task between the retro-cue and memory probe does not affect the retro-cue benefit. Consequently, the retro-cue benefit seems to not depend on sustained perceptual attention (Gao et al., 2022; Hollingworth & Maxcey-Richard, 2013; Rerko et al., 2014). With both types of paradigms, the retro-cue and object-repetition benefits could be due to the strengthening of the binding of the relevant item to its context, providing prolonged accessibility even if the FoA is moved (Oberauer, 2019).

Although research proposes that sustained spatial attention is not needed to maintain a representation in a prioritized state, an unresolved question is whether the selection of this representation within the memory set, to bring it into the FoA, is based on a spatial attentional mechanism, or on the contrary, if the selection of the prioritized representation is independent of visual attention. To shed light into this question, we carried out an EEG while participants performed a combined memory retro-cue task and a perceptual detection task paradigm. We used a retro-cue paradigm because it has provided robust evidence of the internal FoA. This paradigm demonstrates that attention can be directed to a single representation, boosting WM performance, by providing an informative cue during the maintenance period (Astle et al., 2012; Poch et al., 2014; Souza & Oberauer, 2016). To study if the selection of retro-cued representation is accomplished through visual attention, we presented, at an unpredictable onset between retro-cue offset and the memory probe, a stimulus that participants had to detect in the center of the screen. The informativeness of the retro-cue (neutral vs. spatial) and the visual demand of the perceptual attentional task (low-contrast vs. high-contrast stimuli) were also manipulated. With this design, we intended to prevent perceptual switches of spatial attention to the retro-cued representation in the high demanding perceptual condition (low contrast), to test if the retro-cue benefit would be affected. In addition, we explored EEG alpha oscillations, which are considered a robust neural correlate of the locus of the external or internal attention (Kelly et al., 2006; Kuo et al., 2017; LaRocque et al., 2013; van Dijk et al., 2010; van Moorselaar et al., 2015; Woodman et al., 2021). At a behavioral level, we found that the spatial demanding task did not impact the retro-cue benefit. However, the selection of the cued representation did negatively affect the low-contrast detection task. In line with behavioral results, analysis of alpha oscillatory activity revealed that the retro-cue triggered a shift of spatial attention to the cued memory representation, suggesting that perceptual attention is needed to select the relevant WM representation.

## Methods

### Participants

Thirty-one adult subjects (mean age, 24.87 years, standard deviation, 2.36, range 19–29; 16 females) without any history of neurological or psychiatric illness gave written consent, in accordance with the Declaration of Helsinki. Sample size was calculated using G*Power (Faul et al., 2007). With the aim of detecting an effect size of 0.25 and obtaining a statistical power of 0.9 the required sample was for 29 participants.

### Experimental task

**Fig. 1:**
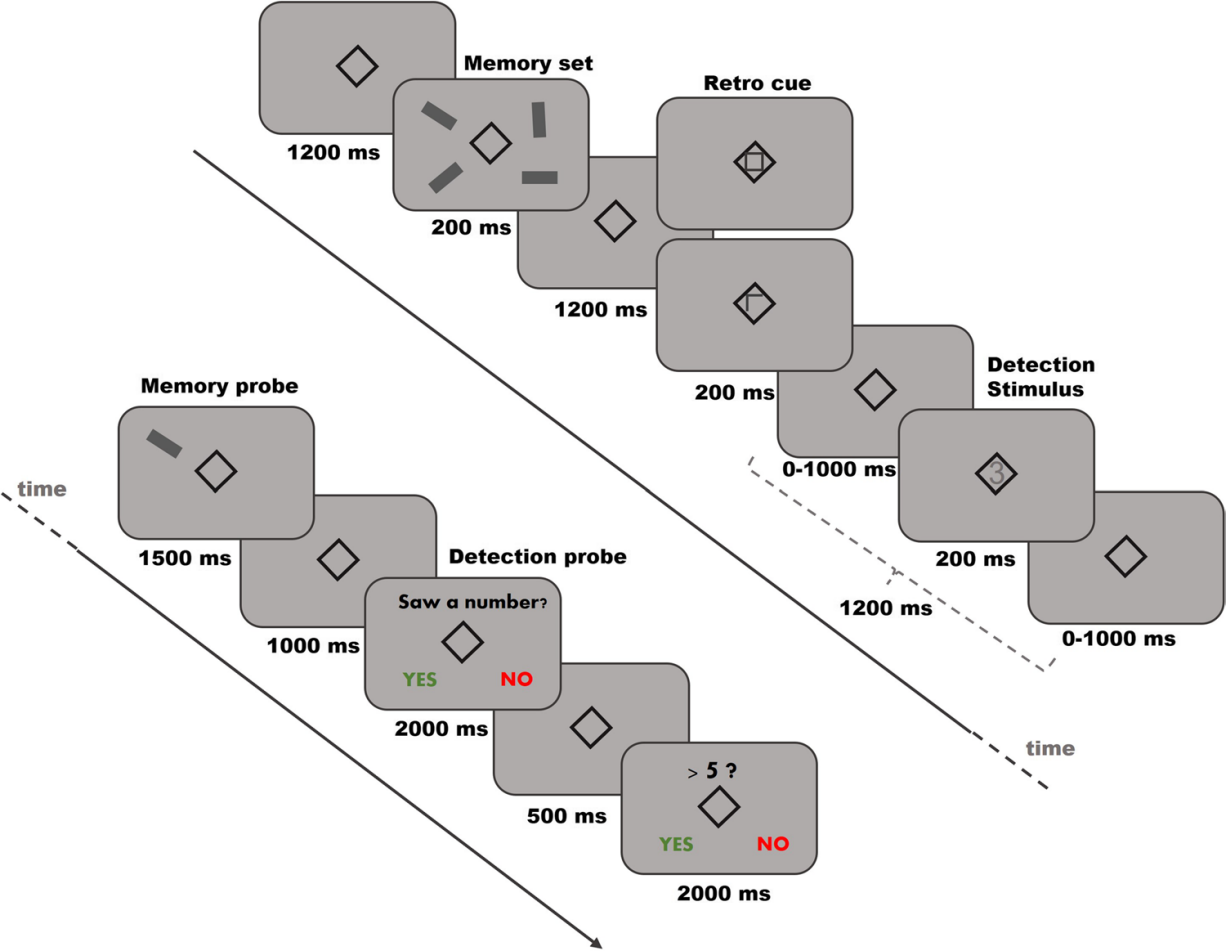
Schematic illustration of the experimental task

The experimental task is illustrated in Fig. 1. A retro-cue WM task was used. The sample memory set consisted of four rectangles, two in each hemifield, with four different orientations and located within 3.8° of visual angle. After a delay interval, participants were presented with either a non-informative cue (neutral cue, 50% of the trials) or an informative cue (spatial retro-cue, 50% of the trials) indicating which rectangle was relevant for posterior testing. After the presentation of the retro-cue, participants were asked to detect a stimulus whose contrast was either near the threshold of perception (low contrast blocks) or clearly visible (high contrast blocks). The probe stimulus was a number inserted in the fixation square (1–9, except 5), randomly presented in the interval of 0–1.2 s after retro-cue presentation. For both types of blocks, the probe was present in 70% of the trials. Perceptual contrast threshold estimation was performed via Palamedes toolbox for MATLAB by means of the Psychometric Function (PF). The contrast value of the probe for the near-threshold blocks corresponded to the 75% value of the PF and was re-calculated during the task depending on the visibility reported by the participants. After this second retention period, participants were presented with a single rectangle and were required to respond whether the orientation of the probe was the same as at encoding. Orientation of the probe rectangle matched that of the relevant sample memory rectangle on 50% of the trials. For non-match trials (50% chance), orientation was randomly selected. Once the participants responded reagrding the memory probe, they had to report whether they had detected the previously presented probe. Additionally, if they saw the probe, there was a 33% chance that they would be asked if the probe was above or below 5. A total of 400 trials, divided into ten blocks of 40, were presented. Participants were informed at the beginning of the block about visibility of the detection stimuli (low or high). The order of the blocks was randomized.

### EEG data recording

EEG data were recorded using a Biosemi Active Two system with 128 electrodes. Additional EOG – vertical and horizontal – electrodes and a nose-tip reference were also recorded. The data were digitized at a sampling rate of 2,048 Hz and low-pass filtered at 410 Hz. Finally, data were re-referenced offline to the nose tip and downsampled to 256 Hz in MATLAB using Fieldtrip (www-fieldtriptoolbox.org). Subsequent preprocessing and analyses were also carried out in Fieldtrip toolbox.

### Preprocessing and oscillatory analyses

Analysis of oscillatory activity was performed in artifact-free data. First, data were epoched in segments from -2.6 to 3 s around retro-cue presentation. Independent Component Analysis (‘runica’ EEGlab implemented in Fieldtrip) was used to extract the vertical and horizontal eye movements out of the signal. Individual epochs were visually inspected to discard epochs with gross artifacts and interpolate noisy electrodes. Alpha oscillatory activity was then calculated using the Hilbert transform. First, artifact-free data were bandpass filtered between 8 and 14 Hz. Then, the spectral amplitude time course of the signal was extracted from the absolute value of the Hilbert transform, and baseline corrected (-300 to 0 pre-memory set). Lateralized alpha activity was calculated by collapsing the left retro-cue condition electrodes with a mirrored version of right condition electrodes. In this way, contralateral activity is represented in right electrodes by averaging right electrodes of the left condition with left electrodes of the right condition. In an analogous way, ipsilateral activity is represented in left electrodes.

### Statistical analyses

WM accuracy was submitted to a 2 x 2 ANOVA with factors Probe-contrast (High and Low contrast) and Cue-type (Spatial cue and Neutral cue). Performance in the perceptual task, in which participants had to indicate if the perceptual probe was present or not, was assessed based on Signal Detection Theory. Signal discriminability and response bias were estimated by the non-parametric indices A’ and B”, respectively (Pallier, 2002). These measures were computed based on Hits and False alarm rates of only correct memory trials. The threshold for declaring statistical significance was α = 0.05.

Differences in alpha oscillatory activity were assessed by means of a non-parametric cluster analysis implemented in Fieldtrip (Maris & Oostenveld, 2007), which controls for Type I error. First, a parametric test is conducted for each electrode-time pair. Then, clusters of significant electrode-time pairs adjacent in time or space are formed. A cluster statistic is computed as the sum of the parametric statistical values forming the cluster, and then tested for significance by comparing it with a null distribution. The permutation distribution is obtained by randomly assigning the data to two subsets and calculating the maximum cluster statistic. A histogram of cluster statistics is obtained by repeating the previous step 10,000 times. Finally, the cluster’s *p*-value is obtained as the proportion of randomizations that are above the observed cluster-level statistic.

## Results

### Memory performance

**Fig. 2:**
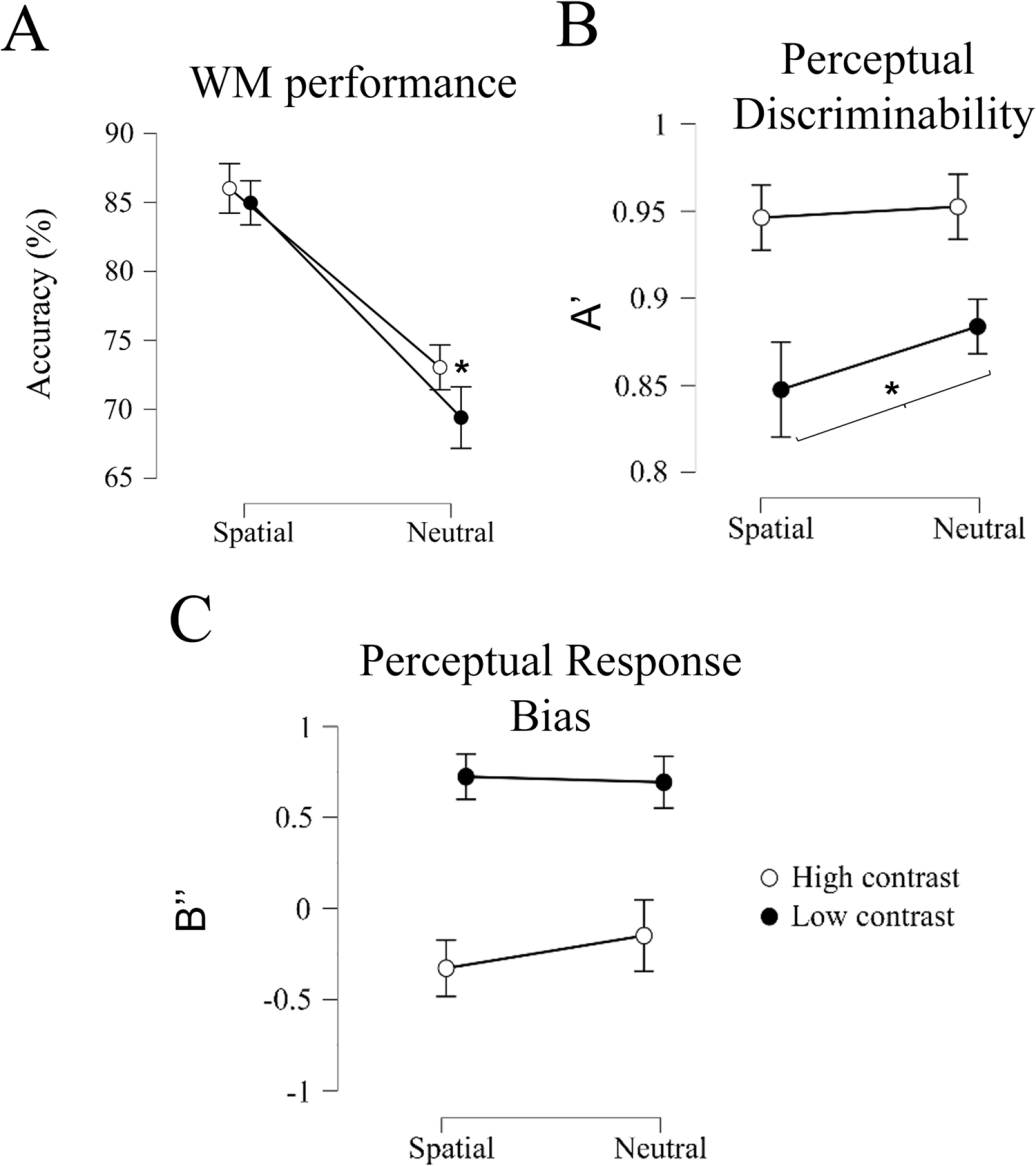
Behavioral performance. The two contrast conditions are represented on different lines. Vertical bars represent 95% confidence intervals. (**A**) Line plot of the percentage of correct responses for the working memory (WM) task. (**B**) Line plot of the A’ values for the detection task. (**C**) Line plot of the B” values for the detection task

Memory performance was modulated by the cue and by the probe contrast. A 2 x 2 repeated-measures ANOVA revealed that memory accuracy was significantly higher for spatial retro-cued trials than for neutral trials (F(1,30) = 176.93, p < 0.001) and for high-contrast probe trials compared to low-contrast probe trials (F(1,30) = 6.41, p = 0.01) (Fig. 2). The significant interaction of both factors (F(1,30) = 4.25, p = 0.04) revealed that probe-contrast affected the two cue conditions differently. While in the neutral condition performance was better for the high-contrast probe trials (*t*(30) = 2.89, p = 0.007; Mdiff = 3.63; Cohen’s *d* = 0.38), there were no differences in accuracy between the two contrast conditions in the spatial retro-cue trials (*t*(30) = 1.09, p = 0.28; Mdiff = 1.05; Cohen’s *d* = 0.08). The retro-cue benefit was found for both contrast conditions (high-contrast condition: (t(30) = 11.61, p < 0.001; Mdiff = 12.97; Cohen’s d = 1.16); low-contrast condition (t(30) = 11.47, p < 0.001; Mdiff = 15.56; Cohen’s d =1.43)).

### Perceptual probe detection performance

Discriminability of the perceptual stimuli was significantly modulated by probe contrast (F(1,30) = 30.756, p < 0.001) and by cue type (F(1,30) = 12.906, p = 0.001), with better performance for the high-contrast condition (t(30) = 6.026, p < 0.001; Mdiff = 0.09; Cohen’s d = 1.20) and neutral cue trials (t(30) = 4.198, p < 0.001; Mdiff = 0.03; Cohen’s d = 1.00). Critically, probe contrast interacted with cue type (F(1,30) = 5.553, p = 0.025), in that discriminability was better for the neutral versus spatial trials in the low-contrast condition (t(30) = -4.173, p < 0.001; Mdiff = -0.03; Cohen’s d = 0.38), but was not significantly different between the two cue conditions in the high-contrast condition (t(30) = -0.723, p = 0.473; Mdiff = -0.009; Cohen’s d = 0.25).^1^

Response bias was modulated by probe contrast (F(1,30) = 107.04, p < 0.001) but not by cue type (F(1,30) = 1.182, p = 0.28). We did not find an interaction between these factors (F(1,30) = 2.379, p = 0.13). Response criterium was more liberal for high-contrast than for low-contrast blocks after both spatial cues (t(30) = -9.230, p < 0.001; Mdiff = -1.05; Cohen’s d = 2.3) and neutral cues (t(30) = -7.392, p < 0.001; Mdiff = 0.84; Cohen’s d = 1.68). There were no differences in response criterium when comparing between spatial and neutral cues either for high-contrast (t(30) = -0.179, p = 0.136; Mdiff = -0.17; Cohen’s d = 0.33) or for low-contrast blocks (t(30) = 0.316, p = 0.753; Mdiff = 0.03; Cohen’s d = 0.07).

### EEG results

**Fig. 3:**
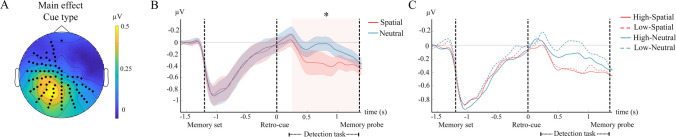
Retro-cued bilateral oscillatory dynamics. (**A**) Topo plot representing the difference of bilateral alpha activity of neutral minus spatial conditions. Alpha activity is collapsed over stimulus contrasts and averaged in the time window conforming the significant cluster. Bold circles represent electrodes making up the significant cluster. (**B**) Averaged alpha time-courses of electrodes making up the significant cluster. Red and blue lines represent spatial and neutral retro-cue conditions, respectively. The orange squared shadow indicates the periods in which statistically significant differences were found. Red and blue shadowed areas indicate the SEM. (**C**) Averaged alpha time-courses of electrodes making up the significant cluster for each condition. Red and blue lines represent spatial and neutral retro-cue conditions, respectively, while solid and dotted lines represent high- and low-contrast conditions, respectively

Non-parametrical statistical analyses revealed that alpha oscillatory activity was modulated by the retro-cue condition, exhibiting higher amplitude in the neutral cue condition than in the spatial cue condition in a cluster of posterior electrodes in the time window from 240 ms until memory probe presentation (*p* < 0.001) (Fig. 3). This load-related alpha power modulation speaks in favor of an effective removal of no-longer-relevant items from WM after the spatial retro-cue. No significant alpha modulation was found between the two contrast conditions, or the interaction between contrast and cue conditions (*p* > 0.05), meaning that the different attentional demands imposed by the expected probe contrast did not modulate anticipatory alpha power.

**Fig. 4:**
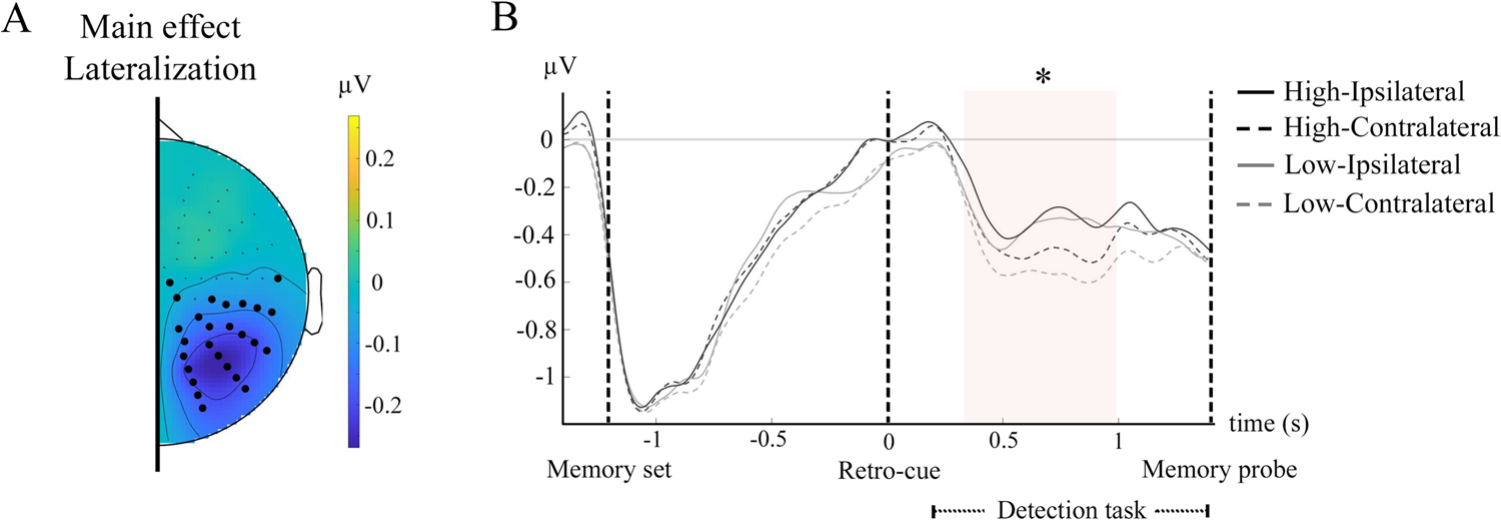
Lateralized alpha activity. (**A**) Topographic representation of the lateralization effect obtained by subtracting contralateral minus ipsilateral activity. Bold circles represent electrodes making up the significant cluster. (**B**) Alpha time-course of contralateral and ipsilateral broken down by stimulus contrast. Black and grey lines represent high- and low-contrast conditions, respectively, while solid and dotted lines represent ipsilateral and contralateral electrodes, respectively. The orange shadow indicates the periods where the main effect of lateralization is significant

Additionally, alpha oscillatory activity was significantly lateralized in spatial retro-cue trials in a cluster of posterior electrodes in the time interval 320–990 ms (p < 0.05). Alpha lateralization is a robust correlate of the direction of spatial attention, so, accordingly, this result indicates that WM prioritization was accomplished through a spatial attentional shift. However, alpha lateralization was not differently modulated by Cue Contrast (p > 0.05) (Fig. 4).

## Discussion

This study investigated the role of perceptual attention in WM selection. We recorded EEG activity while participants performed a retro-cue task. After memory encoding, a spatial (informative) or neutral (non-informative) cue was presented. Participants had to detect a visual stimulus presented randomly during the interval between retro-cue onset and memory probe. Memory performance was compared between blocks with different perceptual attentional demands imposed by manipulating the perceptual contrast of the to-be-detected stimulus. Analysis of WM accuracy revealed a similar retro-cued benefit for the blocks of low- and high-contrast perceptual probe. That is, the higher spatial attentional demand imposed by the low-contrast detection condition did not prevent the prioritization of the retro-cued item. On the other hand, memory accuracy was significantly impaired in the neutral retro-cue condition when the detection task demanded more perceptual attentional resources. The dissociation in WM performance produced by the different perceptual demands in spatial and neutral cued trials was reversed in the visual detection task, with higher detection rates in the neutral cue condition, reflecting a reverse trade-off pattern for the two different representational states.

Previous research has found that engaging in a secondary task that moves attention from memorized items affects memory performance (Awh et al., 1998; Van Der Stigchel et al., 2007). This finding, along with data from eye-tracking and neuroimaging studies, has led to the proposal that memory rehearsal is implemented through a spatial attentional mechanism shared with the perceptual domain. Our results add to this evidence, as WM performance, in the neutral retro-cued trials, was degraded when shifts of perceptual attention were prevented, probably because the spatial rehearsal mechanism was impeded. However, this was not the case for the spatial retro-cue condition, in which we found that the retro-cue benefit was not compromised by the visual detection task. Previous studies have found no evidence of impaired performance when the focus of attention was removed from the relevant WM item (Gao et al., 2022; Hedge et al., 2015; Hollingworth & Maxcey-Richard, 2013; Rerko et al., 2014). Here, despite the fact that we did find an intact retro-cue benefit, we also found a trade-off with detection performance, suggesting that selection within WM implicates a visual attentional mechanism. As opposed to other studies, our secondary attentional task was designed to restrict covert shifts of spatial attention from the offset of the retro-cue, while other studies did not restrict shifts of spatial attention following the retro-cue. Thus, unlike in our study, participants in those experiments could indeed have used visual spatial attention to prioritize a WM item, and then maintained the item in a privileged access status through another retention mechanism not implying sustained visual attention (Hollingworth & Maxcey-Richard, 2013; Muhle-Karbe et al., 2021; Myers et al., 2017). Consistent with this hypothesis, neuroimaging studies using multivariate analysis to decode the contents of WM have found that neural activity tracking the item in the mnemonic FoA dropped to baseline levels with an external shift of attention without affecting memory performance for the memory item (Lewis-Peacock et al., 2012). Nevertheless, the active neural trace could be reactivated by again directing attention to the mnemonic representation.

With respect to the dissociated trade-off pattern found in the spatial versus the neutral cue condition, a reasonable hypothesis is not straightforwardly available. On the one hand, it could be related to participant strategies. Since no instruction was given as to which task was more important, participants could have decided to differently prioritize one of the two tasks, maximizing mean performance. In the case of spatial cued trials, attending to the retro-cued item rather than to the to-be-detected stimuli implied a substantial benefit in memory performance. Another option is that providing a spatial retro-cue automatically triggered attentional orienting. Arrow cues, as human gaze, are important surrounding signals that indicate potential situations that require fast shifts of attention, and consequently have been shown to cause automatic changes of attention (Hietanen et al., 2008; Tipples, 2002). Although it is known that pure symbolic cues, such as color cues, also trigger endogenous retrospective orienting effects (Poch et al., 2017), it is unclear whether arrow cues could additionally trigger automatic orienting to mnemonic representations.

An alternative explanation, suggested by an anonymous reviewer, is that WM selection interferes with perceptual detection by competing for a general resource, in which the process of selecting a WM representation would take precedence. Detection performance in the low-contrast neutral trials would be better, as no selection process is initiated. In the same vein, impaired memory performance in the low-contrast neutral condition would also be explained by a processing difficulty within the task, and not by the pre-emption of the spatial rehearsal mechanism.

In line with the behavioral results, oscillatory power analyses revealed that WM prioritization was in fact accomplished through a spatial attentional shift. Following retro-cue presentation, alpha power decreased in the contralateral visual hemisphere compared to the ipsilateral hemisphere regardless of the attentional condition. Alpha lateralization is a robust correlate of the direction of spatial attention. This neural marker has been repeatedly reported when attention is directed to a visual stimulus, in anticipation of a stimulus, or when attention is directed to a WM item (Capilla et al., 2014; Poch et al., 2014; Schneider et al., 2019; Schneider et al., 2021; Thut et al., 2006; Worden et al., 2000). Similarly, in this study, alpha lateralization indicates that after retro-cue representation the FoA is moved to the spatial position to select the relevant WM item. In line with other findings, alpha lateralization was not maintained through the delay period (Myers et al., 2015; Poch et al., 2017; Poch et al., 2018). In this sense, WM prioritization would be accomplished through a selection mechanism that would strengthen the binding of the memory item to its context with no need of sustained attention (Oberauer, 2019). This is also consistent with behavioral studies supporting the idea that there is no need for sustained attention to maintain representation in a privileged access status (Hedge et al., 2015; Hollingworth & Maxcey-Richard, 2013; Rerko et al., 2014). Van Moorselaar et al. (2018) explored alpha modulations responding to visual and WM demands. Participants had to maintain a spatial location in WM while performing a spatial task. Alpha activity tracked the WM spatial position only until the visual task was presented, at which point alpha activity began to track the content of the external FoA. The alternate tracking of the internal and external FoA by alpha oscillations aligns with the hypothesis of a common attentional mechanism accomplished through neural oscillations (van Moorselaar et al., 2018).

Alpha oscillations are believed to reflect the modulation of internal representations of upcoming events (Di Gregorio et al., 2022; Limbach & Corballis, 2016; Samaha et al., 2017; Samaha et al., 2020). Consequently, modulations of anticipatory alpha power have been shown to correlate with different behavioral parameters of visual perception (van Dijk et al., 2008; Hanslmayr et al., 2007; Lange et al., 2014; Mathewson et al., 2011; Romei et al., 2008; Romei et al., 2010). In this study, the manipulation of the probe’s contrast led to different attentional demands in the visual task. Selective attention enhances neural responsiveness when low-contrast stimuli are processed or expected (Carrasco et al., 2004; Hillyard et al., 1998), and, accordingly, it could be expected that when the low-contrast probe was expected, selective attention would have enhanced sensory neural responsiveness, attenuating alpha power. Considering our behavioral results, this would be the case in the neutral cue condition, in which visual attention to the expected visual probe impaired the maintenance of the whole memory set. However, we found that alpha power did not differ between the two contrast conditions in the detection task, and consequently was not modulated by probe expectation. In the same line of argument, it has been broadly reported that alpha power scales with WM load (Heinz & Johnson, 2017; Poch et al., 2018; Schroeder et al., 2018; Tuladhar et al., 2007). Effectively, here we found lower alpha power after the spatial retro-cue, reflecting a diminished WM load through the effective removal of no-longer-relevant items from WM. It is not clear, however, why alpha power scales with WM load. On the one hand, a prevalent theory links alpha oscillations to the protection of WM representations by the endogenous modulation of distracting input (Bonnefond & Jensen, 2012; Jensen & Mazaheri, 2010). On the other hand, a different view relates alpha oscillations to increasing WM load internal demands (Bollimunta et al., 2008; Palva & Palva, 2007; van Diepen & Mazaheri, 2017). With either view, we should have expected an alpha modulation related to the different attentional demands imposed by the expected probe contrast, either reflecting lower internal processing of WM representations when maintenance was disturbed or reflecting the modulation of visual expectations. In a previous experiment, in which we manipulated the contrast of the retro-cue, we also failed to find alpha modulation upon the expectance of a low-contrast stimulus during WM maintenance (Macedo-Pascual et al., 2022). As in that experiment, the block design could have impacted results due to the lack of variability of expectations within the block. Another possible explanation is that, although behavioral differences in memory performance between the two contrasts in the neutral retro-cue condition were significant, differences in visual assignment resources were not large enough to be detected in the EEG activity.

In sum, this study investigated whether it is possible to prioritize a lateralized WM representation while perceptual attention is needed in an external spatial location. Behavioral results showed an attentional trade-off between visual and WM selection, suggesting, in our view, that the selection of a WM representation could be accomplished through perceptual attentional mechanisms.

## Data Availability

The datasets generated and/or analyzed during the current study are available from the corresponding author on reasonable request.

## Footnotes

1 The same pattern of results was found when including correct and incorrect WM trials.

## Code availability

Custom code used for analyzing the datasets and for stimuli presentation during the current study are available from the corresponding author on reasonable request.
