# Correction to: Selection within working memory impairs perceptual detection

**DOI:** 10.3758/s13423-023-02428-6

**Published:** 2023-12-11

**Authors:** Joaquín Macedo-Pascual, Almudena Capilla, Pablo Campo, José Antonio Hinojosa, Claudia Poch

**Affiliations:** 1 Departamento de Psicología Experimental, Procesos Cognitivos y Logopedia, Universidad Complutense de Madrid, Madrid, Spain; 2 Departamento de Psicología Biológica y de la Salud, Universidad Autónoma de Madrid, Madrid, Spain; 3 Departamento de Psicología Básica, Universidad Autónoma de Madrid, Madrid, Spain; 4 Instituto Pluridisciplinar, Universidad Complutense de Madrid, Madrid, Spain; 5 Centro de Investigación Nebrija en Cognición (CINC), Universidad Nebrija, Madrid, Spain; 6 Departamento de Educación, Universidad Nebrija, C. de Sta. Cruz de Marcenado, 27, 28015 Madrid, Spain

**Correction to: Psychon Bull Rev**

**https://doi.org/10.3758/s13423-022-02238-2**

Joaquín Macedo-Pascual · Almudena Capilla · Pablo Campo3 · José Antonio Hinojosa1,4,5 · Claudia Poch

This paper has been updated with open access.

